# Postpartum Pulmonary Embolism in a Grand Multiparous: A Case Report

**DOI:** 10.7759/cureus.39163

**Published:** 2023-05-17

**Authors:** Jinan S Khalifa, Anjala Nizam

**Affiliations:** 1 Department of Obstetrics and Gynecology, Hatta Hospital, Dubai, ARE; 2 Medical School, Dubai Academic Health Corporation, Dubai, ARE

**Keywords:** disseminated intravascular coagulation, pregnancy, venous thromboembolism, puerperium, pulmonary embolism

## Abstract

A 38-year-old grand multiparous pregnant woman in the United Arab Emirates presented to a secondary hospital in active labor at 38 weeks and two days of pregnancy. She visited the antenatal clinic just once during her entire pregnancy. Antenatally, her venous thromboembolism (VTE) risk assessment score was 2, and she was not started on thromboprophylaxis. Postnatally, she was due to receive a dose of low molecular weight heparin at eight hours postpartum; however, just four hours after delivery, the patient had a cardiac arrest, and it was found by imaging studies that she had a pulmonary embolism. The patient was also found to have disseminated intravascular coagulation, which led to multiorgan failure. The patient passed away two days later. Factors such as a sedentary lifestyle, short inter-pregnancy intervals, and COVID-19 infections could be taken into consideration when screening for VTE risk.

## Introduction

The term venous thromboembolism (VTE) encompasses deep vein thrombosis (DVT) and pulmonary embolisms (PEs), which are both associated with pregnancy and puerperium as women are in a hypercoagulable state in those periods [1]. In both conditions, there is an increased risk of maternal morbidity and mortality [2]. To prevent VTE, women are routinely screened for risk factors both antenatally and postnatally in order to initiate the use of thromboprophylaxis depending on the score they achieve [3].

Here, the authors present a case of a grand multiparous pregnant patient who developed PE and suffered a cardiac arrest just four hours after giving birth during the COVID-19 pandemic despite having an antenatal VTE score of 2.

## Case presentation

**Figure 1:**
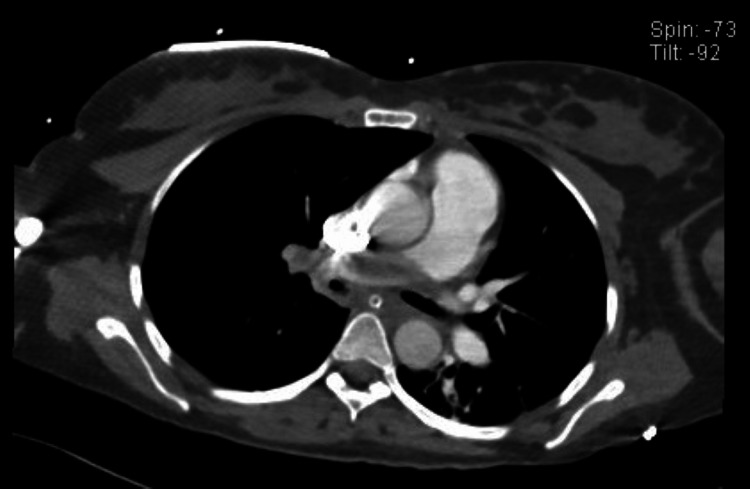
Computed tomography pulmonary angiogram showing a large filling defect at the pulmonary trunk, completely obliterating the right pulmonary artery extending to the proximal left pulmonary artery

A 38-year-old G10P9 pregnant patient was brought to the emergency department of a secondary hospital in June 2020 in the United Arab Emirates (UAE) in active labor at a gestational age of 38 weeks and two days. In the abovementioned pregnancy, she had only one antenatal clinic visit at 37 weeks, as it was during the peak of the COVID-19 pandemic. She had no significant past medical history; however, her past obstetrical history was significant for gestational diabetes managed on diet and pregnancy-induced hypertension in previous pregnancies. Her last childbirth was 16 months ago, and her body mass index (BMI) was 28.19 kg/m^2^. With her age being more than 35 years and her parity being more than three, her antenatal VTE risk assessment score was only 2; therefore, she was not given any thromboprophylaxis. On per vaginal examination in the emergency, it was found that her cervix was 8 cm dilated, membranes were intact, and the head was at −1 station. She was then shifted to the labor suite, where she progressed quickly and delivered her baby, who weighed 2.9 kg. She had an estimated blood loss of 200 mL. The patient was scheduled to receive one prophylactic dose of enoxaparin eight hours postpartum; however, after shifting the patient to the postnatal ward, at approximately four hours postpartum, she suddenly collapsed, became unresponsive, and had a cardiac arrest. Cardiopulmonary resuscitation was then initiated, followed by intubation and ventilation. Return of spontaneous circulation was achieved 40 minutes after the initiation of resuscitation by the multidisciplinary team that included an obstetrician, an anesthetist, a cardiologist, and an intensivist. She was then shifted to the intensive care unit. A computed tomography (CT) pulmonary angiography showed a massive PE that was completely obliterating the right pulmonary artery and minimally extending to the proximal part of the left pulmonary artery (Figure 1).

An initial acute management of thrombolysis therapy was given after her family was counseled. Further thrombolysis was withheld over concerns of disseminated intravascular coagulation (DIC), as evidenced by her labs, as shown in Table 1.

**Table 1:** Laboratory analysis depicting DIC PTT, partial thromboplastin time; DIC, disseminated intravascular coagulation

Parameters	Values	Reference ranges
Platelet count	104 x 10^3^/uL (↓)	150-410 x 10^3^/uL
D-dimer	>4.0 ug/mL (↑)	<0.5 ug/mL
Fibrinogen	31 mg/dL (↓)	200-400 mg/dL
Prothrombin time	>100 seconds (↑)	11-14 seconds
PTT	>180 seconds (↑)	28-41 seconds

**Figure 2:**
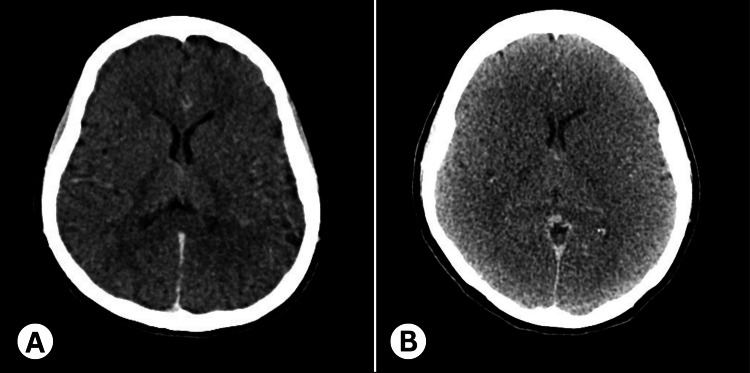
Computed tomography imaging of the brain (A) No evidence of hemorrhage or infarction (B) Global hypoxic-ischemic injury to the brain

A CT scan of the brain was done, which showed no evidence of hemorrhage or infarction (Figure 2A). She was then transferred to a tertiary hospital for further evaluation and possible intervention and management. A follow-up CT scan of the brain done on the same day showed features suggestive of global hypoxic-ischemic injury to the brain, such as an increase in diffuse cerebral edema with partial effacement of gray-white differentiation, effacement of cerebral cortical sulci, and mild compression of the ventricular system (Figure 2B).

Subsequently, she developed acute renal failure, DIC, and multiorgan failure. Unfortunately, she passed away just two days later.

## Discussion

Pregnancy and puerperium are considered to be states of hypercoagulability, which increases the incidence of VTE during these periods [1]. PE is more common in the puerperium than DVT, which is more common antenatally [4]. There are several risk factors for VTE, which are listed in the risk assessment tool for VTE developed by the Royal College of Obstetricians and Gynaecologists [3]. Clinically, acute PE might be difficult to diagnose as symptoms such as dyspnea, chest pain, and tachycardia are common in patients even without PE [5]. Objective diagnosis of PE is mostly done by imaging, which includes CT pulmonary angiography or a ventilation-perfusion scan [6]. Massive PE is known to cause cardiac arrests in patients within a few hours as a result of the development of DIC and subsequent multiorgan failure [7].

The patient mentioned in this case report had several factors that could be attributed to the development of PE in addition to the established risk factors of VTE, which include her advanced maternal age as well as her grand multiparous status. Firstly, the patient had given birth to her last child just six months prior to the onset of the abovementioned pregnancy; hence, she was in a state of continuous hypercoagulability as she had a short inter-pregnancy interval, and this may have contributed to the PE [8]. Secondly, since the patient’s COVID-19 status was unknown and her pregnancy took place during the peak of the pandemic, it is also possible that she could have contracted the SARS-CoV-2 virus at any point during her pregnancy, which would also increase the risk of DVT formation [9]. Lastly, unbooked patients are generally considered to be at high risk of maternal mortality [10]. Though the patient described is not considered an unbooked patient, she only visited the antenatal clinic once in the entire duration of her pregnancy, which may have led to poor antenatal care and hence a factor for maternal mortality in this case.

The population of UAE typically engages in sedentary lifestyles, women more so than men, as reported by a study done by Dalibalta et al. [11]. A few studies have shown that leading a sedentary life can cause the formation of DVTs that may eventually lead to PE [12,13]. Pregnant women are less likely to engage in physical activity, and hence, our patient, in addition to being a UAE resident and leading a sedentary life, being confined to her house due to COVID-19-related restrictions, may have further contributed to decreased physical activity and the eventual PE diagnosis.

## Conclusions

The authors believe that further research must be done to establish if factors such as COVID-19 infection, region-specific sedentary lifestyle, and short inter-pregnancy interval do indeed increase the risk of VTE and necessitate a higher index of suspicion by physicians for DVT and/or PE in such women. Consequently, the antenatal VTE risk assessment tool needs to be updated to reflect these factors.
